# Polytrauma in Older Adults Leads to Significantly Increased TIMP-1 Levels in the Early Posttraumatic Period

**DOI:** 10.1155/2020/4936374

**Published:** 2020-03-09

**Authors:** Mareen Braunstein, Thomas Kusmenkov, Wolf Mutschler, Christian Kammerlander, Wolfgang Böcker, Viktoria Bogner-Flatz

**Affiliations:** ^1^Department of Trauma Surgery, University Hospital Munich, Ludwig-Maximilians University, Nussbaumstr. 20, 80336 Munich, Germany; ^2^Department of Trauma Surgery, Medical University Innsbruck, Anichstrasse 35, 6020 Innsbruck, Austria

## Abstract

**Background:**

Patients after polytrauma regularly suffer from posttraumatic immune system destabilization, which closely influences the further clinical development. Increasing age has recently been identified as an isolated risk factor for an adverse outcome after major trauma. Higher rates and intensity of acute inflammation following severe injury suggest that deregulated inflammation may contribute to these higher rates of posttraumatic morbidity and mortality in older adults. MMP-9 and TIMP-1 have been found to play a major role in posttraumatic immune disorder in a previous genome-wide mRNA analysis.

**Objective:**

The aim of this study was to evaluate the differences in serum protein dynamics in older and younger polytraumatized adults.

**Methods:**

Blood samples were drawn immediately within 90 minutes after trauma and subsequently after 6, 12, 24, 48, and 72 h. Serum levels of TIMP-1 and MMP-9 were quantified using ELISA. Age groups were divided according to a cutoff of 60 years.

**Results:**

60 polytrauma patients (ISS > 16) were included (<60 years, *n* = 49; ≥60 years, *n* = 49; ≥60 years, *n* = 11). Serum TIMP-1 and MMP-9 levels showed a highly significant serum dynamic in young and old polytrauma patients (*p* < 0.001). Patients ≥ 60 years showed significantly higher overall TIMP-1 levels (*p* < 0.001). Patients ≥ 60 years showed significantly higher overall TIMP-1 levels (*p* = 0.008). TIMP-1 levels showed a significant maximum after 72 h in the older study population. MMP-9 levels were nonsignificantly higher during the whole observational period in older polytrauma patients when compared to younger patients.

**Conclusion:**

The posttraumatic immune response is characterized by significantly higher TIMP-1 levels in older polytrauma patients. This significant association between TIMP-1 levels and patients' age indicates a more extensive immune dysregulation following major trauma in older adults.

## 1. Introduction

Systemic immune activation and dysfunction are still a major cause of late posttraumatic morbidity and mortality [1, 2]. Of course, this is true for patients of all ages, but growing evidence suggests that especially older polytrauma patients are at risk to develop posttraumatic complications. Recent studies have documented mortality rates of more than double in this population [3]. The presence of comorbidities and adaptive changes that occur as a natural process of aging reduces the physiological reserves and compensatory capacity thereby affecting trauma outcomes. Further, higher rates and intensity of trauma-induced systemic inflammation seem to contribute to posttraumatic morbidity and mortality following severe injury in this cohort. A previous systematic review reported remarkably higher incidences of systemic inflammation in older surgical patients [4]. 1-month mortality after SIRS and septic shock among older patients was 10% and 40–60%, respectively [5, 6]. Irrespective of major trauma, aging is associated with several physical changes including the innate immunity leading to severe dysregulation of initiation, modulation, and determination of the primary inflammatory response [7]. This phenomenon is known as “inflamm-aging” and characterized by high plasma levels of circulating proinflammatory cytokines [8, 9]. Considering these facts, the combination of age-related and trauma-related immune dysregulation might contribute to higher posttraumatic morbidity and mortality in older adults [10]. However, only little is known about posttraumatic systemic immune response of older polytrauma patients, although their number is increasing consequently [3]. Previous genome-wide studies have linked specific mRNA expression patterns in monocytes with adverse outcome. Among these differentially expressed genes, matrix metalloproteinase-9 (MMP-9) and its specific tissue inhibitor-1 (TIMP-1) could be identified to play an important role in trauma patients in the early posttraumatic period. Both have shown a differential higher expression depending on injury severity and 90-day survival after major trauma [11]. Further, both are implicated in various aspects of inflammation including accumulation of inflammatory cells, healing of tissue injury, and remodeling processes. MMP-9 is a type IV collagenase and stored in the tertiary granules of polymorphonuclear leucocytes, which are key effectors in acute inflammatory diseases. Specifically, it has been shown to mediate vascular leakage and to initiate the migration of inflammatory cells. TIMP-1 works as a natural inhibitor of MMP-9 and is found in most tissues and body fluids. By inhibiting MMP activities, TIMPs are involved in tissue remodeling and regulation of ECM metabolism. Under normal physiological conditions, TIMPs bind MMPs in a 1 : 1 stoichiometry. Consequently, a loss of activity control may result in a variety of inflammatory diseases. Thus, the balance of MMP and TIMP activities plays the pivotal role in both physiological and pathological events. The aim of the present study was to evaluate the differences in serum protein dynamics in older and younger polytraumatized adults in order to recognize the underlying immunological changes in the early posttraumatic period.

## 2. Materials

### 2.1. Study Setting and Population

After permission by the Regional Ethical Review Board of Ludwig-Maximilians University, Munich, Germany (reference number: 012/00), the study was performed at our Level I trauma center following Good Clinical Practice. A consent form of study participation was obtained from the patients or a legal representative. Patients (≥18 years) suffering from blunt multiple injuries with an Injury Severity Score (ISS) of more than 16 points were included. Only patients who were admitted to our emergency department within 90 minutes after trauma were enrolled. Main exclusion criteria were acute infectious disease, immunosuppressive therapy, terminal disease, or pregnancy. Patients' treatment was performed according to standard of care and was not affected by study participation. After the initial resuscitation and/or primary surgical interventions, patients were admitted to the surgical intensive care unit. Patients who died within the first 24 h after trauma were excluded from the study. The following variables were recorded for each patient: demographic data, clinical parameters and comorbidities, and duration of stay in the intensive care unit and in the hospital. According to their age, patients were stratified into two groups. The definition of older patients used in this current work refers to patients ≥ 60 years. This prospective study was designed to correlate TIMP-1 and MMP-9 serum concentrations with patients' age in order to gain insight into age-dependent posttraumatic systemic immune disorders.

### 2.2. Blood Sampling and Protein Levels

Blood samples were drawn within the first 90 minutes after trauma on admission (0 h) and 6 h, 12 h, 24 h, 48 h, and 72 h after trauma. Sampling time points were strictly standardized to the traumatic event. Afterwards, samples were stored at -80°C. Protein concentrations were quantified by enzyme-linked immunosorbent assay (ELISA; Human MMP-9 and Human-TIMP-1 ELISA, Bender MedSystems GmbH). For the analysis of MMP-9 and TIMP-1 protein levels in serum, sample dilutions of approximately 1 : 100 to 1 : 500 for MMP-9 and 1 : 1000 to 1 : 5000 for TIMP-1 were required.

### 2.3. Statistical Analyses

Statistical analyses were performed using SPSS Statistics Version 24.0 (IBM Corporation; Armonk, NY, USA). To take sequential measurements into account and to evaluate protein dynamics in particular, we selected a statistical model for repeated measures analyses (repeated measures MANOVA, SPSS General Linear Model). The following independent parameters were included in the model: “time” to indicate significant protein dynamics and “age.” Consequently, we could investigate differences in the overall protein levels, detect dynamic protein changes over time, and identify the influence of age on the type and extent of protein changes. If findings were statistically significant, the single points in time were secondarily evaluated using nonparametric Mann-Whitney *U*-test. Descriptive statistical analyses (Table 1) were performed using chi square and Mann-Whitney *U*-test setting *p* < 0.05 as the level of significance.

## 3. Results

### 3.1. Clinical Baseline Characteristics

**Figure 1:**
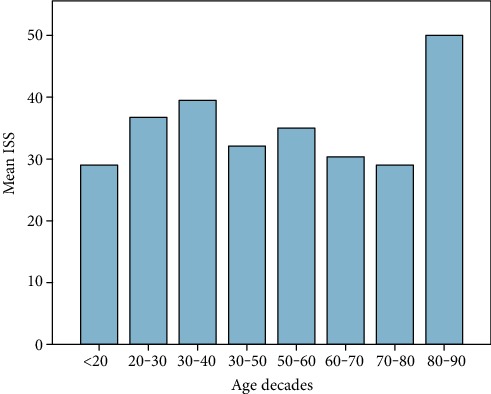
Mean ISS of all patients according to age decades (*n* = 60). The results show higher ISS scores between 20 and 40 years and over the age of 80 years.

**Figure 2:**
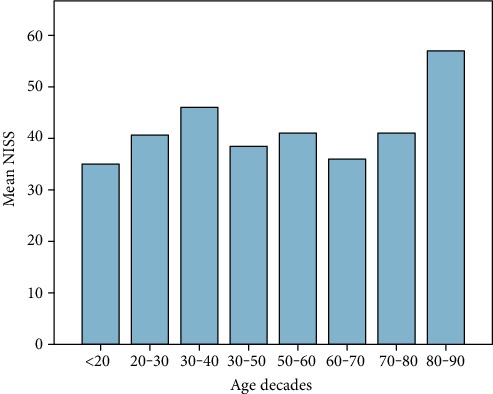
Mean NISS of all patients according to age decades (*n* = 60). The results show higher NISS scores in patients aged between 20 and 40 years and again between the ages of 70 and 90 years.

**Figure 3:**
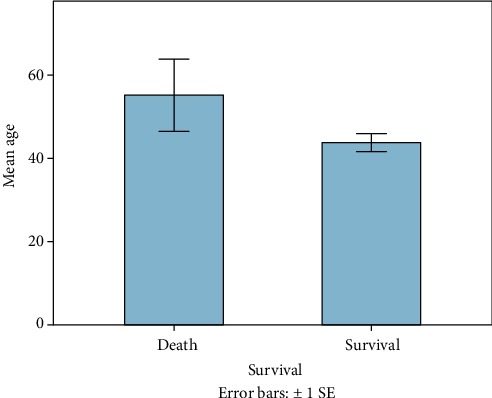
Outcome according to patient's mean age. Patients who died after the traumatic event were older on average when compared to younger polytrauma patients (nonsurvivors *n* = 8, mean age 54.9 years; survivors *n* = 52, mean age 43.4 years).

**Figure 4:**
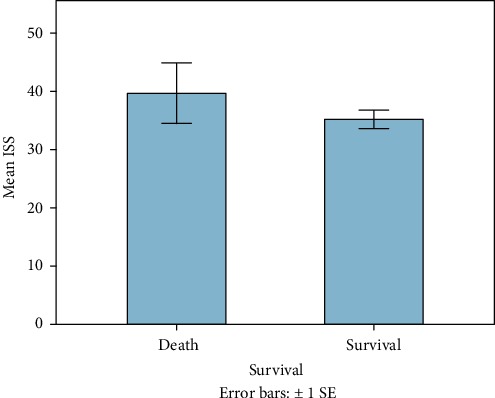
Mean ISS of all patients correlated to patient's outcome. Patients who died after the traumatic event showed higher mean ISS than those who survived (mean ISS survivors: 35 ± 11.6 SD; mean ISS nonsurvivors: 39.3 ± 12.2 SD).

**Figure 5:**
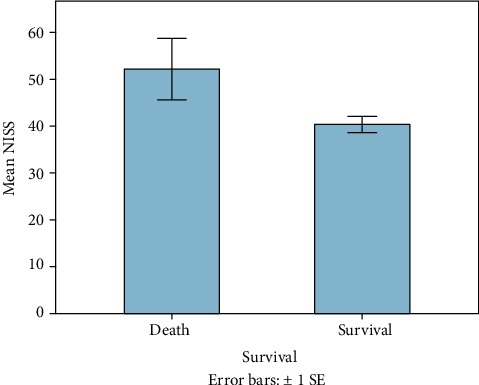
Mean NISS of all patients according to patient's outcome. Patients who died after the traumatic event showed higher mean NISS than those who survived (mean NISS survivors: 40.2 ± 12.8 SD; mean NISS nonsurvivors: 50.5 ± 12.2 SD).

60 patients fulfilled the entry criteria and were included in the presented study. Mean age was 45 years ranging from 18 to 93 (±16.6 SD, median 44.5). 70% were male (*n* = 42), and 18 patients were female (30%). Mean ISS of all patients was 35.6 (±11.7SD, median 34, range: 17-66), and mean NISS was 41.5 (±13.7SD, median 41, range: 17-75). Mean GCS was 10 (±4.7SD, median 11.5). Mean ISS and mean NISS according to age decades are illustrated in Figures 1 and 2. 13% of patients did not survive the observational period (24 h-90 days; survivors *n* = 52; nonsurvivors *n* = 8). Survivors' mean age was 43.4 years (±16SD, median 42, range 18-84), and 38 patients were male (73%). Nonsurvivors were 54.9 years old (±18.2 SD, median 50, range: 37-94), and 50% were male. 90-day survival according to mean age of all patients is illustrated in Figure 3. Mean ISS of all survivors was 35 (±11.6 SD, median 32, range: 17-66), and mean ISS of nonsurvivors was 39.3 (±12.6 SD, median 44, range: 17-50) (Figure 4). NISS of all survivors ranged from 17 to 75 (mean ISS 40.2 ± 12.8 SD, median 39.5), and mean NISS was 50.5 (±12.2 SD, median 57, range: 33-75) in the group of nonsurvivors (Figure 5).

Patients were divided into two groups regarding their age with a cutoff value of 60 years (<60 years, *n* = 49; ≥60 years, *n* = 11). Mean age in patients < 60 years was 39.2 years (±11.5 SD, median 38, range: 18-59), and 35 were male (71%). Patients presented with a mean ISS of 36 (±12 SD, median 35, range: 17-66) and a mean NISS of 41.5 (±14.2 SD, median 41, range: 17-75). Mean GCS was 9.9 (±4.8SD, median 11). Six patients < 60 years did not survive the observation period of 90 days. Mean age of nonsurvivors < 60 years was 47 years (±8.1 SD, median 48.5), and three were male. Nonsurvivors presented with a mean ISS of 41.2 (±9.7 SD, median 44, range: 26-50) and a mean NISS of 52.2 (±16.1SD, median 67, range: 33-75). Mean age of all patients ≥ 60 years was 70.5 (±10.8 SD, median 65, range: 60-94), and seven were male (64%). They showed a mean ISS of 33.8 (±10.8 SD, median 29, range: 17-50) and a mean NISS of 40.2 (±11.4 SD, median 34, range: 27-50). Patients ≥ 60 years presented with a mean GCS of 10.2 (±5 SD, median 13). Two patients ≥ 60 years did not survive the observation period (mean age 78.5, one male). Nonsurvivors had a mean ISS of 33.5 (±23.3 SD) and a mean NISS of 45.5 (±16.3 SD). Groups were comparable with regard to gender (male < 60 years: 71.4%; male ≥ 60 years: 63.6%), trauma severity (ISS < 60 years: 36, ISS ≥ 60 years: 34), and incidence of traumatic brain injury (TBI < 60 years: 46.9%; TBI ≥ 60 years: 45.5%). Detailed clinical data of all included patients are depicted in Table 2.

Statistical analysis of all patients' characteristics are depicted in Table 1. Regarding injury severity, patients < 60 years showed significantly more injuries to the extremities than older patients did (AIS ≥ 3 extremities/pelvic girdle; *p* = 0.029). Regarding all other items, both groups were comparable.

### 3.2. Serum Protein Concentrations

#### 3.2.1. TIMP-1

**Figure 6:**
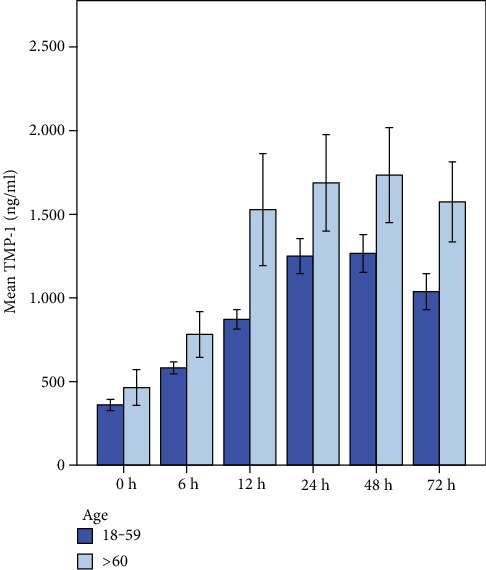
TIMP-1 serum levels within the first 72 h after trauma correlated to patient's age. Multivariate testing of TIMP-1 serum levels and time after trauma revealed a highly statistical significance (*p* < 0.001) in both groups. TIMP-1 levels were significantly higher in patients ≥ 60 years when compared to younger polytrauma patients (*p* = 0.008). Post hoc analysis revealed significantly higher TIMP-1 levels in older patients after 72 h (*p* = 0.039).

TIMP-1 serum concentration levels within the first 72 h after trauma are illustrated in Figure 6. Multivariate testing of TIMP-1 serum levels and “time” after trauma revealed a highly statistical significance (*p* < 0.001). In detail, TIMP-1 levels of patients younger than 60 years showed a highly significant increase of TIMP-1 concentration after trauma (*p* < 0.001; mean values are given in ng/ml ± SD: 0 h: 357.2 ng/ml (±218.6), 6 h: 583.2.6 ng/ml (±234.8), 12 h: 901.2 ng/ml (±404.6), 24 h: 1254.2 ng/ml (±703.2), 48 h: 1240.4 ng/ml (±142.5), and 72 h: 1090.8 ng/ml (±722.9)). Older polytrauma patients also showed a highly significant increase of TIMP-1 over the time course of 72 h after trauma on a generally higher level (*p* < 0.001; mean values: 0 h: 464.7 ng/ml (±355.7), 6 h: 781.6 ng/ml (±453.5), 12 h: 1528.3 ng/ml (±1109.1), 24 h: 1687.6 ng/ml (±954.9), 48 h: 1733.9 ng/ml (±943.1), and 72 h: 1574.3 ng/ml (±795.7)). The general linear regression model (test of between-subject effects) revealed a significant correlation between mean TIMP-1 serum levels and “age.” TIMP-1 levels were significantly higher in patients over the age of 60 when compared to younger polytrauma patients (*p* = 0.008). Post hoc analysis revealed significantly higher TIMP-1 levels in older patients (≥60 years) when compared to the younger patients' sample 72 h after polytrauma (*p* = 0.039).

#### 3.2.2. MMP-9

**Figure 7:**
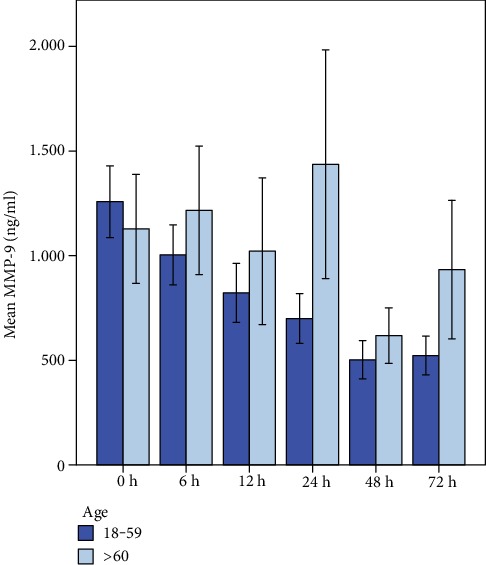
MMP-9 serum levels over the first 72 h after trauma correlated to patient's age. Multivariate analysis of concentration kinetic of MMP-9 and time after trauma revealed a highly statistical significance (*p* < 0.001) in both groups. General linear regression model (test of between-subject effects) revealed no statistical significance for MMP-9 serum levels and age (*p* = 0.227).

MMP-9 serum concentration levels over the first 72 h after trauma are illustrated in Figure 7. Multivariate analysis of concentration kinetic of MMP-9 and “time” after trauma revealed a highly statistical significance for both groups (*p* < 0.001). In detail, mean values (ng/ml ± SD) of patients < 60 years showed a significant decrease over the course of 72 h (0 h: 1282.1 ng/ml (±1086.2), 6 h: 1080.3 (±913.3), 12 h: 814.5 (±808.8), 24 h: 685.7 (±684.4), 48 h: 532 (±528.5), 72 h: 513.5 (±544.6)). Concentration kinetic of MMP-9 showed a different time course revealing higher levels in patients older than 60 years beginning 6 h after trauma (0 h: 1411.7 (±1223.5), 6 h: 12161.4 (±1052.7), 12 h: 1016.9 (±1052.7), 24 h: 1338.6 (±1673.3), 48 h: 620.6 (±398.5), and 72 h: 955.2 (±995.6)). The general linear regression model revealed no statistical significance for MMP-9 serum levels and “age” (*p* = 0.227).

## 4. Discussion

To our knowledge, this is the first study reporting on TIMP-1 and MMP-9 serum levels in older polytrauma patients in the early posttraumatic period. Major trauma in older adults becomes more frequent due to an increasing life expectancy and activity in this population [12]. A retrospective study recently documented that up to 14% of severely injured patients were over the age of 65 years when they were admitted to the emergency department [13]. This is comparable to our findings with 18% of polytraumatized patients being older than 60 years. Due to a missing definition of “geriatric polytrauma,” we defined a cutoff value of 60 years in our study. A recent survey showed that several age definitions were used for “geriatric trauma patients” varying from 55 to 80 years [12, 14]. The working group on multiple trauma of the German Trauma Society found that mortality in polytrauma patients significantly increased already beginning at the age of 56 years—independent of the degree of injury [15]. Increasing age has been identified as an isolated risk factor for adverse outcome after major trauma [15]. Finelli et al. found that patients over the age of 65 years had markedly higher mortality (27% vs. 14%) and complication rates following major trauma when compared to younger polytrauma patients [16]. Giannoudis et al. even found a mortality rate of 42% in the older population [13]. Nevertheless, the understanding of the underlying mechanisms leading to these dramatically high mortality rates in geriatric polytrauma patients is still limited. The role of higher incidence of preexisting comorbidities, the reduced physiological reserves, and compensatory mechanisms secondary to aging seem apparent. Until today, it is not clear if other factors further contribute to a worse outcome in older trauma patients [15]. Therefore, it seems increasingly important to define the association between age and polytrauma outcome. In this context, the posttraumatic systemic inflammatory reaction might play an important role. A recent study could show that older polytraumatized patients more often develop a posttraumatic SIRS than younger patients [17]. Several studies have noted that older patients have increased rates and intensity of acute inflammation following severe injury suggesting that trauma-induced deregulated inflammation may contribute to these higher rates of morbidity and mortality in the elders [4, 18]. Considering these facts and the key role of MMPs and their inhibitors in posttraumatic immune disorder as identified in our precedent genome study, we consequently evaluated TIMP-1 and MMP-9 in older polytrauma patients in order to elucidate the particular inflammatory immune reaction in this vulnerable population. The most relevant and new findings of this study were the following: TIMP-1 and MMP-9 concentrations demonstrated a significant dynamic over the first 72 h after major trauma in older polytrauma patients. TIMP-1 showed a significant correlation between serum levels and age, as overall TIMP-1 levels were significantly higher in patients over the age of 60 years. In contrast to younger polytrauma patients, the increase of TIMP-1 levels did not influence MMP-9 levels in the older adults. Contrary, posttraumatic immune response was accompanied by higher MMP-9 levels in the older group, whereas MMP-9 levels significantly decreased in younger patients. When regarding single time points in the early posttraumatic course, TIMP-1 levels were significantly higher at 72 h after polytrauma in older patients. The important role of both proteins following tissue injury and acute inflammation—as induced by several pathological conditions such as major trauma, sepsis, burn, and severe TBI—has recently been confirmed by several clinical studies [19–21]. Lorente and coworkers found a significant increase of TIMP-1, and nonsignificantly higher MMP-9 levels, in sepsis patients, and both proteins were significantly higher in nonsurvivors [22]. TIMP-1 was elevated in burn patients depending on the severity of the burn, and higher TIMP-1 levels were independently associated with 90-day mortality [23]. Higher TIMP-1 levels were associated with increased mortality rates after traumatic brain injury presenting a prognostic biomarker in TBI patients [24]. In particular, TIMP-1 seems to work as an indicator for the severity of pathological immune activation and is associated with worse outcome in these cases.

When interpreting our results, the main question to answer is whether higher levels of the detected proteins are simply the result of the “normal aging process” characterized by increasing inflammatory mediators or whether it is the result of the traumatic impact leading to a more severe immune activation in older patients. The knowledge about age-related changes of serum MMPs and their inhibitors is important to avoid misinterpretation of data. Few studies have examined the effect of age on MMP and TIMP levels resulting in conflicting data [25–28]. Bonnema et al. documented that TIMP-1 plasma levels increased significantly as a function of increasing age, whereas MMP-9 levels decreased [26]. In turn, Beaudeux et al. and Manicourt et al. did not reveal increasing TIMP-1 serum levels depending on increasing age [27, 28]. Another study stated a negative correlation between TIMP-1 and aging without any correlation between MMP-9 and age [29]. Regarding these data, increased levels of TIMP-1 and MMP-9 in our cohort more likely reflect the pronounced immune reaction after trauma in older patients than simply the physiological aging processes. These results seem to be relevant as increased levels of inflammatory mediators have been shown to act as predictors of mortality independent of preexisting morbidity in older patients [9]. Recently, Jylhä and colleagues found that higher blood levels of CRP, IL-6, and IL-1ra were able to predict mortality in patients of very old age [30]. This state of “chronic inflammation” was found to contribute to the pathophysiology of many age-related diseases like atherosclerosis, diabetes, and neurodegenerative disorders [9, 31]. Interestingly, inflammatory mediators such as CRP, TNF-*α*, IL-1ra, IL-6, IL-8, and IL-10 have been identified to be part of both: age-related diseases and trauma-induced immune dysfunction [9, 32, 33]. In conclusion, the quantity of the involved inflammatory markers seems to contribute to the extensive posttraumatic inflammatory response in the elders more likely than their quality. The same mediators are involved in musculoskeletal changes in older adults such as sarcopenia, frailty, and functional decline [34]. Particularly, TNF-*α* and IL-6 have been associated with muscle loss in older persons [35]. Chung et al. have identified an upregulation of proinflammatory cytokines such as IL-6, IL-*β*, and TNF-*α* and have documented a potential underlying molecular mechanism, namely, the NF-*κ*B-dependent network which is associated with numerous age-related diseases including dementia, cardiovascular disease, and osteoporosis [36]. This is especially interesting in regard to our results, because MMP-9 contains a nuclear factor-*κ*B site, which is activated during inflammatory processes as seen in traumatic brain injuries [37]. Transferring the abovementioned data to our cohort, significantly higher TIMP-1 levels in the older population reflect a more severe immune activation following major trauma indicating an interaction between inflammatory markers in case of aging and polytrauma. Our data might give a hint that these patients are potentially at risk to develop posttraumatic complications due to this more pronounced immune dysregulation. Presumably, damage control surgery might be even more important in this vulnerable population in order to reduce the additive physiological load (“second hit”) and cellular stress of an extensive surgical procedure [12]. However, only little is known about this population until today, and there is a specific need for additional research in this area to further reduce posttraumatic morbidity and mortality in this trauma population.

## 5. Limitations

A main limitation of the presented work is the relatively small sample size especially in the older population. As a result, we could only reliably report on the trauma-associated protein level changes indicating that the patient's age seems to play an important role particularly when regarding TIMP-1 level changes. Unfortunately, a further correlation to the development of multiple organ failure or mortality was not possible as the group of older polytrauma patients was too small. Although, it is known that larger study populations are difficult to achieve in this population, as they suffer from polytrauma less often, the small sample weakens the significance of our study. Furthermore, our study lacks a control group to investigate the baseline level of TIMP-1 especially in the elderly which might have strengthen our results. Nevertheless, our findings justify a larger study assessing the value of TIMP-1 as a potential prognostic biomarker in older polytrauma patients.

## 6. Conclusion

Age-related chronic inflammation is characterized by an overstimulation of the innate immune system called “inflamm-aging.” It is accompanied by high levels of proinflammatory markers and considered to be one of the most important contributors to morbidity and mortality. Additional cellular stress as induced by severe polytrauma results in a state of “hyperinflammation” presented by higher TIMP-1 and MMP-9 levels. Our data present an important immunological process following major trauma in this vulnerable population and might thereby help to improve the understanding of the posttraumatic inflammatory immune response in geriatric trauma patients. Evaluating MMP-9 and TIMP-1 changes in a follow-up study in order to show the impact of these changes on patient's outcome might help to identify “patients at risk” and thereby prevent or at least delay functional decline in older polytrauma patients by adapting further treatment strategies to the special need of this population.

## Figures and Tables

**Table 1:** Statistical analysis of patients' characteristics (Mann-Whitney *U*-test, chi square; *p* < 0.05).

Patient characteristics	Total	Patients < 60 years	Patients ≥ 60 years	*p* value
Patients (*n*)	60	49 (82%)	11 (18%)	
Age (mean ± SD)	44.9 ± 16.6	39.2 ± 11.5	70.5 ± 10.8	
Gender (male/female)	42 (70%)/18	35 (71%)/14	7 (64%)/4	0.610
ISS (mean ± SD)	35.6 ± 11.7	36.0 ± 12.0	33.8 ± 10.8	0.604
NISS (mean ± SD)	41.5 ± 13.7	41.5 ± 14.2	40.2 ± 11.4	0.830
AIS head/neck ≥ 3 (*n*)	30 (50%)	23 (47%)	7 (64%)	0.317
AIS face ≥ 3 (*n*)	3 (5%)	2 (4%)	1 (9%)	0.491
AIS chest ≥ 3 (*n*)	49 (82%)	39 (80%)	10 (91%)	0.381
AIS abdominal/pelvic contents ≥ 3 (*n*)	20 (33%)	16 (33%)	4 (36%)	0.831
AIS extremities/pelvic girdle ≥ 3 (*n*)	34 (57%)	31 (63%)	3 (27%)	0.029^∗^
AIS external ≥ 3 (*n*)	2 (3%)	2 (4%)	0	0.633
GCS (mean ± SD)	10.0 ± 4.7	9.9 ± 4.8	10.2 ± 5.0	0.892
TBI (*n*)	28 (47%)	23 (47%)	5 (45%)	0.929
MOV (*n*)	52 (87%)	43 (88%)	9 (82%)	0.601
90-day survival (*n*)	52 (87%)	43 (88%)	9 (82%)	0.601
Surgery/24 h after trauma	24 (40%)	18 (37%)	6 (55%)	0.276

**Table 2:** Detailed patients' characteristics including gender, age, injury severity, initial GCS value, and survival (*n* = 60). Patients are divided into two groups according to the cutoff age of 60 years.

Patient	Gender	Age	ISS	NISS	GCS	Survival
<60 years						
1	f	32	66	66	11	Y
2	m	18	36	48	7	Y
3	f	24	22	27	14	Y
4	m	33	34	75	14	Y
5	m	20	22	22	15	Y
6	f	25	50	57	5	Y
7	m	33	34	34	5	Y
8	f	24	38	38	15	Y
9	m	23	43	43	15	Y
10	f	35	34	34	6	Y
11	m	30	45	57	3	Y
12	m	34	59	59	4	Y
13	m	29	41	41	15	Y
14	m	34	66	66	3	Y
15	m	29	29	34	7	Y
16	m	23	41	41	3	Y
17	f	23	25	34	3	Y
18	m	32	35	43	15	Y
19	f	28	34	34	3	Y
20	m	23	36	41	3	Y
21	m	53	36	41	11	Y
22	f	54	25	29	7	Y
23	m	49	18	27	6	Y
24	m	49	17	17	15	Y
25	m	44	17	17	15	Y
26	m	53	36	48	3	Y
27	f	37	43	75	15	N
28	m	40	29	29	11	Y
29	m	58	29	29	15	Y
30	m	49	26	34	13	N
31	m	45	20	34	15	Y
32	m	46	36	48	15	Y
33	m	36	29	34	13	Y
34	m	48	41	41	7	Y
35	f	57	22	27	15	Y
36	m	44	57	57	6	Y
37	m	37	43	50	13	Y
38	m	51	29	41	8	Y
39	m	37	29	34	15	Y
40	m	59	50	57	15	N
41	m	51	29	34	6	Y
42	f	48	35	43	15	Y
43	m	38	33	33	13	N
44	m	49	36	48	4	Y
45	m	57	57	57	13	Y
46	f	51	45	57	6	N
47	m	39	24	24	14	Y
48	f	40	34	34	12	Y
49	f	48	50	57	7	N

≥60 years						
50	f	74	29	29	14	Y
51	m	62	27	27	15	Y
52	m	75	24	29	15	Y
53	f	63	17	34	15	N
54	m	94	50	57	3	N
55	m	65	45	57	8	Y
56	f	61	50	50	13	Y
57	f	65	34	34	3	Y
58	m	60	29	34	14	Y
59	m	84	29	41	4	Y
60	m	72	38	50	9	Y

Note: f: female; GCS: Glasgow Coma Scale; ISS: Injury Severity Score; m: male; NISS: New Injury Severity Score; N: no; Y: yes.

## Data Availability

Full data will be made available on request.
